# MR1-restricted T cells: the new dawn of cancer immunotherapy

**DOI:** 10.1042/BSR20202962

**Published:** 2020-11-13

**Authors:** Zhiding Wang, Mengzhen Wang, Jinghong Chen, Linlin Zhang, Li Zhang, Li Yu

**Affiliations:** 1 Department of Hematology and Oncology, International Cancer Center, Shenzhen University General Hospital, Shenzhen University Health Science Center, Shenzhen 518000, China; 2 Department of Hematology and BMT Center, Chinese PLA General Hospital, Beijing 100853, China

**Keywords:** Cancer, Immunotherapy, MAIT, MR1-restricted T cell, MR1

## Abstract

Cancer immunotherapy has recently undergone rapid development into a validated therapy for clinical use. The adoptive transfer of engineered autologous T cells, such as chimeric antigen receptor (CAR) T cells, has been remarkably successful in patients with leukemia and lymphoma with cluster of differentiation (CD)19 expression. Because of the higher number of antigen choices and reduced incidence of cytokine release syndrome (CRS) than CAR-T cells, T cell receptor (TCR)-T cells are also considered a promising immunotherapy. More therapeutic targets for other cancers need to be explored due to the human leukocyte antigen (HLA)-restricted recognition of TCR-T. Major histocompatibility complex (MHC), class I-related (MR1)-restricted T cells can recognize metabolites presented by MR1 in the context of host cells infected with pathogens. MR1 is expressed by all types of human cells. Recent studies have shown that one clone of a MR1-restricted T (MR1-T) cell can recognize many types of cancer cells without HLA-restriction. These studies provide additional information on MR1-T cells for cancer immunotherapy. This review describes the complexity of MR1-T cell TCR in diseases and the future of cancer immunotherapy.

## Introduction

Major histocompatibility complex (MHC) class I-related (MR1) is a major histocompatibility complex (MHC)-like molecule that is conserved across species and humans [1]. The MR1 gene locates on chromosome band 1q25.3, has 4 isoforms, and is ubiquitously expressed in all tissues. MR1 was originally reported in the presentation of bacteria antigens to mucosal-associated invariant T (MAIT) cells. MAIT cells are an evolutionarily conserved sub-population of T cells in mammals, characterized by the expression of the semi-invariant TCRα (TRAV1-2/TRAJ33), which restricts to the non-polymorphic MR1 [2–4]. Unlike the more well-known cluster of differentiation (CD)4^+^ and CD8^+^ T cells, MAIT cells can recognize riboflavin-derivative antigens through conserved T-cell receptors (TCRs), which is not restricted by human leukocyte antigen (HLA) [5]. The MR1-T cell TCR repertoire and its presentation is quite complex and differ in cancers, infections, and other diseases, and has not been fully explored [6]. Studies have found that MR1 with presentation antigen can be recognized by some MR1-T cell clones in cancers and could be a promising target of cancer immunotherapy [7,8]. Compared with chimeric antigen receptor (CAR)-T and TCR-T cells, MR1-T cells present additional advantages may have greater potential in immunotherapy.

### MR1

TCR recognition is vital to the body as a defense mechanism against infection and tumors. The TCR can recognize peptide antigens, as well as a wide range of other antigens. Unlike the conventional MHC-I and MHC-II molecules that have been extensively studied, some MHC-like molecules (e.g., HLA-E, CD1, and MR1) have been attracting increasing attention in terms of antigen presentation [9]. HLA-E, CD1, and MR1 present non-self-lipids, peptides, and metabolites separately that could be recognized by TCR, indicating that T cells have additional roles in immune responses to tissue homeostasis, inflammation, infection, and cancer [10].

HLA-E is a homologous MHC class Ib molecule that is widely expressed at low levels and can present peptides derived from MHC class Ia molecules (VMAPRTLVL, VL9) to the natural killer (NK) cells where they bind to cognate receptor molecules like the NK cell lectin-like receptor C1/2 (NKG2A/C)–CD94 complex [11]. The CD1 is for lipid antigen presentation through its CD1a, CD1b, CD1c, and CD1d isotypes. CD1a, CD1b, and CD1c are expressed by thymocytes, dendritic cells (DCs), mononuclear phagocytes, and other cells [12]. MR1 is a ubiquitously expressed non-polymorphic MHC class I-like molecule, which presents a family of bacteria-derived metabolites to MAIT cells.

MR1 is characterized by three α domains, a transmembrane domain, and a cytoplasmic tail. Analysis of the crystal structure of MR1 shows that it associates with β-2-microglobulin at the α3 domain [13]. The TCR α chain plays a more important role than the β chain in MR1 recognition. X-ray studies have shown that the complementarity-determining region (CDR) 1 and CDR2 loops of the TCR α and β chains straddle the α1 and α2 helices of MR1, placing the invariant CDR3α loop in a conserved position of the MR1 antigen-binding pocket [14].

MR1 antigen presentation commonly occurs in various bacterial and yeast infections, but not in viral infections, which were first found to be sensed by MAIT cells [4]. Derivatives of 5-amino-6-d-ribitylaminouracil (5-A-RU), the product of an intermediate in the riboflavin biosynthesis pathway, have been detected by the TCR of MAIT cells. 5-A-RU can produce 5-(2-oxopropylideneamino)-6-d-ribitylaminouracil (5-OP-RU) and 5-(2-oxoethylideneamino)-6-d-ribitylaminouracil (5-OE-RU) derivative antigens by enzymatic reactions with methylglyoxal or glyoxal [15]. MR1 [5,16] can bind to additional ligands such as hesperidin, diclofenac, aspirin analog 3-formylsalicylic acid (3-FSA), methotrexate derivative 2,4-diamino-6-formylpteridine (2,4-DA-6-FP), riboflavin, riboflavin-derived photolumazine I (PLI), PLIII, and the riboflavin analog 7,8-didemethyl-8-hydroxy-5-deazariboflavin (FO). 3-FSA and 2,4-DA-6-FP can be competitive inhibitors of MAIT cell function [16,17]. There is also evidence that tumor-related antigens exist that can bind to MR1 and active MAIT cells, although the specific antigens from tumors are yet to be identified [7,18].

### MAIT cells in tumor

MAIT belongs to a population of unconventional T cells that play important roles in the human immune system, including responses to bacterial and viral infection diseases, autoimmune disease, cancer, allergies, and inflammatory disorders [6]. Current T-cell studies mainly focus on the more well-known CD4^+^ and CD8^+^ T cells. Other T cells with the capability of recognizing non-peptide antigen presentation have not been reported to date. CD8^+^ and CD4^+^ T cells can recognize short peptide antigens presented by MHC class I (8–11 aa) and MHC class II (15 aa) antigens [9]. MAIT cells can recognize riboflavin-derivative antigens presented by MR1 [19,20]. Unlike CD4^+^ and CD8^+^ T cells that possess diverse TCR repertoires for polymorphic HLA structures, MAIT cells have a quite conserved TCR repertoire, which is not restricted by HLA. In humans, MAIT cells constitute 10% of blood T cells and 45% of liver T cells [21–23]. The first MAIT cell TCRα (TRAV1-2/TRAJ33) sequence was revealed in 1993 [24]. By 1999, MAIT cell TCR repertoire conservation between mammalian species and its unique sequence were reported [25]. Subsequent studies have shown that MAIT cells are restricted to MR1, and TRAV1 that co-evolved with MR1 was also highly conserved [1]. The activation of MAIT cells leads to migration and expansion with cytokines [26,27], which is similar to the activity of other T cells. MAIT cells can be activated by the TCR and pathogen-associated molecular pattern/pattern recognition receptor (PAMP) [6], and in turn can activate DC and NK cells [28]. MAIT cell subsets are now typically defined by the expression of the TCR chain. The classical MAIT cell expresses the TCR α-chain is TRAV1-2^+^ TRAJ33^+^, with two alternative TRAJ genes (*TRAJ12* and *TRAJ20*) [29]. It is required to discover deeply for other TRAV1-2^−^ MAIT cells such as non-classical MAIT cells (TRAV36^+^ TRBV28^+^ with CD161, CD218, CD26, and PLZF expression) and atypical MR1-restricted T cells [30].

MAIT cells can act directly against a range of bacterial and fungal species via the riboflavin-biosynthesis pathway, even against some cell-invading intracellular bacteria and parasites [6]. Because viruses are incapable of synthesizing riboflavin, the response of MAIT cells to viruses is dependent on cytokines [31]. MAIT cells also play a part in autoimmune disease and inflammatory disorders and many other diseases [6]. However, the mechanism underlying their activity remains unclear.

Previous studies have described the role of MAIT cells in tumor immunity. A meta-analysis of expression signatures from ∼18000 human tumors with overall survival outcomes across 39 malignancies found that KLRB1 (encoding CD161) expression is a favorable prognostic gene, which is also a marker of MAIT cells [32]. Colorectal cancer specimens showed that MAIT cells gather around tumor lesions from the peripheral blood [33]. Another study showed that the circulation of peripheral blood MAIT cells occurred in mucosal (gastric, colon, or lung) tumors or cervical cancer [34], but did not occur in non-mucosal (breast, liver, or thyroid) tumors.

MAIT cells also play a role in hematological malignancies. More exhausted MAIT cells are detected in newly diagnosed multiple myeloma (MM) patients and are also restored in refractory or relapsed patients. MM patients have few MAIT cells in blood and bone marrow compared with healthy subjects [35]. MAIT cells from healthy individuals can kill MM cell lines through MR1 with 5-OP-RU. In addition, Langerhans cell histiocytosis patients also show that MAIT cells gather around tumor lesions from peripheral blood [36]. MAIT cell-deficient mice Mr1^–/–^ that received allogeneic bone marrow transplantation develop more severe graft-versus-host disease (GVHD) than normal mice [37]. A lower number of MAIT cells contributed to a higher risk of delayed acute GVHD and reduced overall survival [38].

### MR1-T cell in diseases

MR1-T cells are not only TRAV1-2^+^ MAIT cells, but other TRAV1-2^−^MR1-T cells. The diversity of antigens’ specificities of these TRAV1-2^−^ MR1-T cells indicates a new area for future studies. The discovery of novel MR1-T cell subsets and distinct antigens reflects the structural diversity of MR1 presentation, which could be more diverse than 5-OP-RU and 6-FP (Table 2). Additional studies on MR1-restricted antigens and the TCR repertoire may provide new insights into tumor immunity and other diseases [39].

## Bacterial infections

Meermeier et al. [40] demonstrated the presence of a new functional human MR1-T cell clone exhibiting TRAV12-2/TRAJ39 and TRBV29-1/TRBJ1-5 expression. This is a novel pattern of microbial recognition by detecting infection with *Streptococcus pyogenes*, a pathogen that lacks the riboflavin synthesizing ability. It is suggested that the TRAV12-2 clone recognizes unique antigens and might play a specific role in the defense against infection by broadening the recognition of microbial metabolites, which shows that MR1-T cells have the capacity to detect a greater diversity of microbes than previously shown. Howson et al. [23] found a diverse TRBV repertoire from TRAV1-2^+^ T cells by utilizing samples from a controlled human infection model of the invasive bacterial pathogen *Salmonella paratyphi A* in healthy volunteers, and specific infection-expanded TRAV1-2^+^ with TRBV6-1/TRBJ2-3 transfection T cells can sense *Escherichia coli* and *Salmonella paratyphi A* culture supernatants.

However, whether MAIT cells discriminate between many species of the human microbiota remains unclear. Tastan et al. [41] developed an *in vitro* functional assay using human T cells engineered for MAIT-TCRs stimulated by MR1-expressing antigen-presenting cells (APCs). They then screened 47 microbiota-associated bacterial species from different phyla and found that only bacterial species that encoded the riboflavin pathway were stimulatory to MAIT-TCRs. Most species that were high-stimulators belonged to the phyla Bacteroidetes and Proteobacteria, whereas low/non-stimulator species were primarily Actinobacteria or Firmicutes. The activation of MAIT cells by high- vs*.* low-stimulating bacteria was also correlated with the level of riboflavin they secreted or after bacterial infection of macrophages. There is a highly significant difference among bacterial species in terms of their MAIT-TCR stimulatory capacity. Furthermore, T cells can act as APC for antigen-specific activation of MAIT cells and tuning of their effector functions. Huang et al. [42] developed a method that allowed the selection of rare cells to study antigen-specific T-cell clonality. The authors used SELECT-seq to collect both TCR sequences and then transcriptomes from single cells of peripheral blood lymphocytes activated by a *Mycobacterium tuberculosis* lysate. TCR sequence analysis allowed the authors to preferentially select expanded conventional CD8^+^ T cells as well as invariant NK T cells and MAIT cells.

## Cancer

Lepore et al. [7] found an atypical MR1-restricted T cell clone (DGB129) that did not react to microbial ligand-recognized cancer cell lines (CCRF-SB, THP-1, and A375-MR1). Lepore et al. [7] also showed that this MR1-restricted T cell clone DGB129 (TRAV29/TRAJ23 and TRBV12-4/TRBJ1-1) can respond to MR1 in the absence of microbial antigens and can recognize cancer cells (leukemia and melanoma cell lines) through interactions with MR1 molecules produced by the cancer cells. The cells can easily be detected in the blood of healthy individuals and were classified as a new cell population based on their capacity to recognize MR1 and on their ability to react to different types of cancer cells. Importantly, no significant differences in how MR1 recognizes these TCRs in individuals was observed, although the TCR may recognize MR1-expressing cancer cells from different patients. Although the nature of these molecules remains to be determined, the initial characterization of the molecules showed that these formed stable complexes with a plastic-bound MR1 without forming a Schiff base and activated specific MR1T cells without the need for APC processing.

Crowther et al. [8] found a single TCR (MC.7.G5, TRAV38-2/TRAJ31, and TRBV25-1/TRBJ2-3) that can recognize and kill many human cancer types via MR1, but not normal cells. MR1-restricted T cells made from the MC.7.G5 clone can kill a broad range of cancer cells regardless of HLA. MC.7.G5 MR1-restricted T cells can also kill leukemia cells *in vivo* and prolong the survival of mice. In addition, MC.7.G5 transferred patient T cells can kill autologous and non-autologous melanoma cells.

## Other diseases

Contentti et al. [39] identified a wide range of TRBV repertoires from TRAV1-2^+^ T cells of volunteers with multiple sclerosis. By recognizing different antigens occurring in distinct target cells and exhibiting a variety of effector functions, the MR1-restricted T cells have been shown to drive inflammatory responses, support B-cell function, mediate DC licensing, promote tissue remodeling, and contribute to the maintenance of mucosal homeostasis by enhancing innate defenses at the epithelial barrier. Therefore, future studies are expected to uncover the roles of MR1-restricted T cells in other human diseases.

### MR1-T cells TCR repertoire

Numerous studies have shown that MR1-T cells are more diverse than MAIT ‘invariant’ cells. The diversity of the MR1 repertoire is complex and associated with different antigens, suggesting that MR1-T cells exhibit more functions than expected [7,43] (Table 1).

**Table 1:** MR1-restricted T cells TCR repertoire

Resource	TCR usage				Stuats	Sample type	Donor (*n*)	Year
	TRAV	TRAJ	TRBV	TRBJ				
Porcelli et al. [24]	TRAV1-2	TRAJ33	TRBV6 family		Healthy	Blood	5	1993
			TRBV20-1					
Tilloy et al. [25]	TRAV1-2	TRAJ33	TRBV6		Healthy	Blood	3	1999
			TRBV20					
Reantragoon et al. [29]*	TRAV1-2	TRAJ33	TRBV6-4		Healthy	Blood	6	2013
		TRAJ12	TRBV20					
		TRAJ20						
Gherardin et al. [44]^†^	TRAV1-2	TRAJ33	TRBV6	TRBJ2	Healthy	Blood	5	2016
		TRAJ12	TRBV4	TRBJ1-5				
		TRAJ20	TRBV20-1					
			TRBV28		MM		1	
			TRBV5					
Voillet et al. [45]	TRAV1-2		TRBV6		Healthy	Blood, lymph	4	2018
			TRBV4					
			TRBV20-1					
			TRBV2					
			TRBV3-1					
Koay, et al. [30]^‡^	TRAV36	TRAJ34	TRBV28	TRBJ2-5	Healthy	Blood	4	2019
		TRAJ37	TRBV5-1					

* More TCR. ^†^Some atypical TCRs. ^‡^More TRAV1-2^−^ TRAV36^−^ TCRs.

**Table 2:** MR1-restricted T cell in diseases

Resource	TCR usage				Clone	Year	Function
	TRAV	TRAJ	TRBV	TRBJ			
Meermeier et al. [40]	TRAV12-2	TRAJ39	TRBV29-1	TRBJ1-5	D462-E4	2016	*S. pyogenes*
Lepore et al. [7]*	TRAV29	TRAJ23	TRBV12-4	TRBJ1-1	DGB129	2017	Leukemia and melanoma cells
Howson et al. [23]*	TRAV1-2		TRBV6-1	TRBJ2-3		2018	*S. paratyphi* A
			TRBV6-4	TRBJ2-1			
Tastan et al. [41]	TRAV1-2	TRAJ33	TRBV20	TRBJ2-1		2019	Bacteroidetes and Proteobacteria phyla
			TRBV2				
			TRBV13				
			TRBV12				
Contentti et al. [39]	TRAV1-2		TRBV20-1	TRBJ1-2		2019	Multiple sclerosis
			TRBV6	TRBJ2-1			
			TRBV7-9	TRBJ1-5			
			TRBV7-6	TRBJ2-7			
			TRBV14	TRBJ1-3			
Huang et al. [42]	TRAV1-2	TRAJ33	TRBV6-4	TRBJ1-1		2019	*M. tuberculosis*
				TRBJ2-2			
				TRBJ2-1			
Crowther et al. [8]	TRAV38-2	TRAJ31	TRBV25-1	TRBJ2-3	MC.7.G5	2020	Tumor and leukemia

* More TCR

Porcelli et al. [24] first detected an enriched TCR α-chain TRAV1-2/TRAJ33 CD4^−^CD8^−^ double-negative T cell and subsequently an invariant TCR α-chain paired with a constrained TCR β-chain repertoire (TRBV6 family and TRBV20-1) in humans. In 2013, Reantragoon et al. [29] found that not all the MAIT TCR α-chains paired with TRAV1-2 and TRAJ33. There are also MAIT TCRs that use TRAJ12 or TRAJ20 instead of TRAJ33 in pairing with TRAV1-2. Gherardin et al. [44] found that the TCR β-chain repertoire not only belongs to the TRBV6 and TRBV20-1 family, but also to the TRBV4, TRBV28, TRBV5, TRBJ2, and TRBJ1-5 families [23]. Identified human T cells can utilize distinct TRAV-TRAJ-TRBV genes to generate MR1-restricted TCRs with different antigen recognition patterns [7]. Some of these cells are capable of recognizing MR1-5-OP-RU, whereas others recognize only 6-FP and/or Ac-6-FP. Therefore, MR1-T cells exhibit diversity in TCR gene usage and antigen specificity. Voillet et al. [45] also found that the TCR β-chain repertoire includes TRBV2 and TRBV3-1.

Koay et al. [30] reported that human TRAV1-2^−^ MR1-restricted T cells contain both MAIT-like and non-MAIT-like cells. These include a MAIT-like population that expresses a public canonical TRAV36/TRAJ34^+^ or TRAJ37^+^ TRBV28/TRBJ2-5^+^ TCR. These TCRs utilize a different docking strategy to the TCRs of classical MAIT cells and thus may also permit recognition of distinct antigens beyond 5-OP-RU.

### CAR and TCR T-cell cancer immunotherapy

CAR-T has been proven to be therapeutically effective in patients with CD19^+^ B cell leukemia and lymphoma [46]. Based on remarkable response rates, autologous CD19-targeted CAR T cells became the first to be used in genetically engineered immunotherapy approved by the United States Food and Drug Administration [47]. However, there are still some major obstacles for CAR-T cells in cancer immunotherapy. First, the therapeutic function of CAR-T cells is not good enough for solid tumors [48]. Second, it is difficult to identify a specific target antigen similar to CD19, which is expressed on the surface of cancer cells. Third, cytokine release syndrome (CRS) and neurotoxicity develop when CAR-T cells are overactivated.

TCR-T cell TCR chains have an antigen-binding region, an extracellular constant region, and a transmembrane domain. The TCR chain transduces an intrinsic signal, which is dependent on T-cell accessory signaling molecules to induce T-cell activation. TCR-T cell immunotherapy presents some advantages over CAR-T cell immunotherapy. First, TCR-T cell immunotherapy clinical trials indicate that this approach is more efficacious against solid tumors. Second, TCRs can recognize peptide epitopes derived from any subcellular (e.g., membrane, cytoplasm, and nucleus) compartment protein [49]. Third, TCR chains possess an intrinsic signal and regulate T cells, thus resulting in a lower risk for the development of CRS and neurotoxicity.

**Figure 1:**
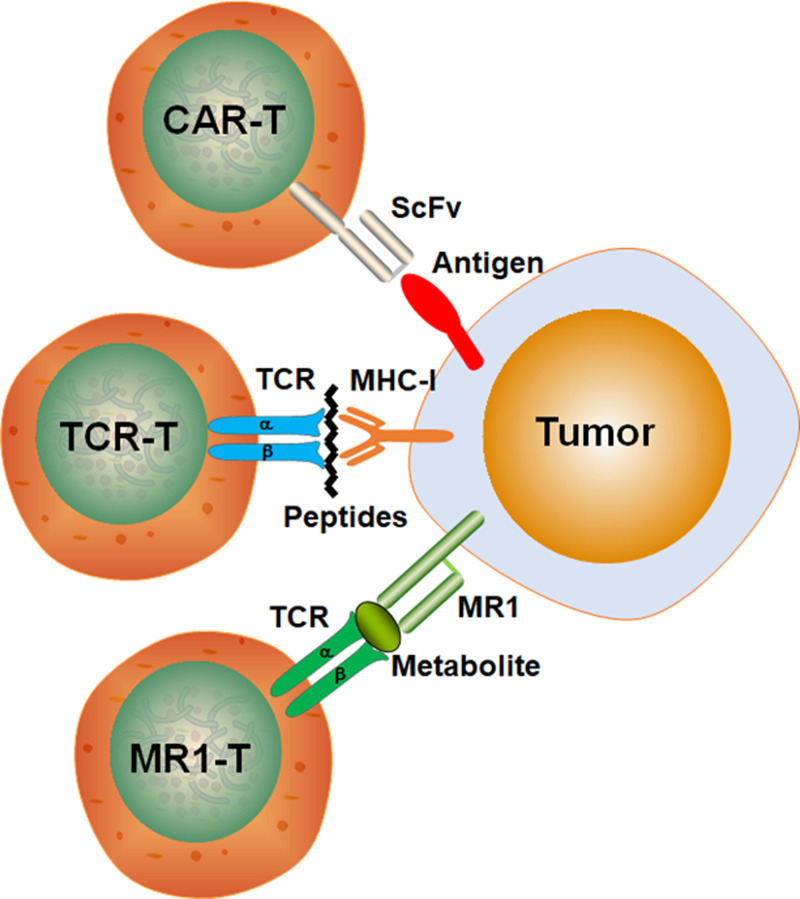
Schematic diagram of CAR-T, TCR-T, and MR1-T immunotherapy tumor recognition

The CAR-T and TCR-T cell immunotherapies also have some limitations (Figure 1). TCRs can potentially recognize peptides presented by HLA molecules, which allow TCRs to target intracellular antigens (e.g., neoantigens, cancer germline antigens, and viral oncoproteins). However, TCR-T cell immunotherapy can only recognize targets expressing the designed HLA proteins, thereby restricting its utility to patients. Furthermore, cancers can down-regulate MHC and other antigens to immunologically escape recognition by CAR-T and CAR-T cells [50].

### MR1-T cell cancer immunotherapy

MR1-T cells can target many kinds of cancer cells but not healthy cells, using a single TCR clone [8]. Unlike CAR and TCR T cell immunotherapy that require different targets for various cancers (Figure 1), MR1-T cells can recognize and kill tumor cells via one target and one TCR clone.

MR1T cells (clone DGB129) can recognize human leukemia (CCRF-SB and THP1) and melanoma (A375-MR1) cancer cell lines through MR1 presentation *in vitro* [7]. Lepore et al. found that MR1-T cells can recognize antigens bound to MR1. To identify the antigens of MR1 presented during tumor recognition, THP-1 total cell lysate supernatant was collected and separated by C18 high performance liquid chromatography. Different MR1-T cell clones (DGB70 and DGB129) recognize different THP-1 cell lysate fractions co-cultured with THP-1 (*in vitro*) and the mouse breast tumor cell line EMT6 (*in vivo*). Thus, the MR1 can present different antigens and be recognized by different TCR clones. In addition, various T cell clones show differential transcriptomes and are functionally heterogeneous after stimulation [7]. The antigens that MR1 presents are complex and remain unknown.

Another MR1-T cell (clone MC.7.G5) [8] was identified in HLA-mismatched healthy donor cells co-cultured with A549 lung cancer cells, and its MR1 target was confirmed using CRISPR-Cas9 lentivirus libraries. This clone can kill lung (A549), melanoma (FM-45, MEL 624, MM909.11, MM909.20, MM909.21, and MM909.24), leukemia (Jurkat, K562, and MOLT3), myeloma (U266), colon (COLO 205), breast (MCF-7), prostate (LnCAP), renal (RCC17 and HEK293T), bone (U-2 OS), and ovarian (A2780 and EOC031) cancer cell lines and MM primary cells *in vitro*. This clone does not kill normal tissues or cells such as lung fibroblasts, hepatocytes, and alveolar, intestinal, renal, pancreatic, melanocyte, smooth muscle, bronchial, dendritic, and Langerhans cells. Moreover, this clone is safe *in vitro* when tested with whole peripheral blood mononuclear cells (PBMCs) and resting or activated purified T and B cells of healthy donors, and in normal renal epithelial cells under tert-butyl hydroperoxide (tBHP) and H_2_O_2_ stress or γ irradiation. Even healthy lung epithelial cells are safe with *Mycobacterium smegmatis* infection. This MR1-T cell (clone MC.7.G5) could kill Jurkat cells in NSG mice and prolong survival. Two metastatic melanoma patient-derived T cells transduced with the TCR of MC.7.G5 can recognize autologous and non-autologous melanomas, but not primary healthy melanocytes or pancreatic and lung cells. Thus, these MR1-T cells may be utilized as a universal tool to safely target cancer cells.

A well-studied and functional MR1-T cell clone can be utilized in humans without HLA-restriction. MR1 in mammals is highly conserved [1] compared with classical MHC-I genes, as indicated by a much slower rate of evolution/genetic changes. MR1 is not subjected to pathogen-induced selection because it is not evolved in antigen presentation. TRAV genes are very large and have multiple subgroups, and they show a wide range of variations. However, TRAV1 mainly interacts with MR1 and is highly conserved. This is quite rare in contrast with most other TRAV subgroups, which interact with polymorphic classical MHC-I. Compared with other TRAV genes, the TRAV1 and the TRAJ33 used by MAIT cells is highly conserved. MR1 is not only conserved across species but also is non-polymorphic across different ethnicities [9]. MR1 is a ubiquitously expressed MHC class I-like molecule that is expressed on all types of human cells. These unique characteristics make MR1 an ideal target for killing various kinds of tumors using a few MR1-T cell clones.

MR1-T cell immunotherapy can be safe for clinical use. MR1-T cells utilize the TCR structure to find tumors and activate an intrinsic signal similar to that of TCR-T cell immunotherapy. The intrinsic signal organizes antigen receptor signaling to amplify and coordinate changes in T cell function and induces cytokine secretion [49]. Unlike CAR-T cells that exhibit signaling via punctate accumulation of Lck, which is a rapid and transient downstream signal transduction pathway, MR1-T cells may act similar to TCR-T cells, which initiate downstream signaling via a non-covalent complex with a CD3ζζ homodimer, CD3δε, and CD3γε heterodimers, and a total of ten immunoreceptor tyrosine-based activation motifs (ITAMs). CAR-mediated killing appears to be more rapidly attenuated than TCR-mediated killing. MR1-T cells act via TCR recognition thus is more similar to actual T-cell activation in the body with less CRS.

## Future and perspectives

MR1-T cells have no restrictions in target finding, and new TCR clones can easily be established. The CAR targeting structures are based on single-chain variable fragment (scFv), and its ligand primarily recognizes cell surface antigens (e.g., unmodified proteins, glycoproteins, glycolipids, and carbohydrates) in an HLA-independent manner. Only ∼27% of the human proteome is represented on the surface of cells and recognized by CAR. The TCR recognizes epitopes derived from the entire proteome, although it still needs to be presented via the HLA. This restricts the utility of TCR-T cell immunotherapy, as presentation peptides continue to change with HLA types. Due to proteome complexity, cancer heterogeneity, gene mutation, epigenetic modification, and oncogene fusion, presentation peptides are too short (8–11 amino acids) to be considered unique. These antigen presentation and recognition problems of CAR-T and TCR-T cells could be easily resolved by MR1-T cells [49]. Because MR1-T cells can recognize cancer regardless of cancer type and HLA diversity, finding one effective MR1-T cell could be universally utilized in all cancer patients [8]. This also resolves research challenges relating to complicated antigens and in finding recognition structures.

Based on the above advantages, allogeneic MR1-T cells could be easily manufactured and utilized off-the-shelf [51]. off-the-shelf allogeneic MR1-T cells have many potential advantages over autologous MR1-T cells because of the immediate treatment of cryopreserved cells from donors, which can be standardized and industrialized. Cell modification is generally time-consuming and requires clinical redosing or a combination of different immunotherapies. Like CAR-T cells, the MR1-T cell could be more powerful in combination with chemotherapy, checkpoint inhibitors, epigenetic therapy and cytokines. Therefore, some issues require further investigation.

The therapeutic MR1-restricted TCR α and β transfer to T cells could undergo mispairing with the original TCR and generate heterodimers. These heterodimers do not undergo thymic selection and thus could be recognized as normal antigens and result in severe GVHD. This could be resolved by knocking out the original TCR α or β chains and expressing only therapeutic TCR [49]. Crowther et al. [8] found a MR1-restricted TCR clone MC.7.G5 that can recognize and kill most human cancer types via MR1. This finding indicates that it may be utilized as a universal MR1-T cell clone and thus should be given greater attention. Interestingly, this MC.7.G5 clone recognizes cancer cells, whose metabolome differs from that used for recognition of bacteria by MAIT cells. Cancer recognition suggests that this metabolic pathway is essential and unique to cancer cells. However, the actual ligand of the MC.7.G5 clone has not yet been identified. Additional TCR clones and ligands would be useful, and thus future studies should focus on this point. The discovery of MR1-restricted ligands may also pave the way for new vaccines. MR1-T cells may represent a new hope for cancer immunotherapy with conserved and universal expression profiles. An off-the-shelf MR1-T cell immunotherapy could be established for all cancers based on the role of MR1 in the immune system, and these may also find application in infection and autoimmune diseases.

Although the use of MR1-T cells as a novel immunotherapy strategy for cancer has several advantages, several issues need to be resolved. First, the MR1 antigen presentation mechanism remains largely unknown, including the metabolism products that MR1 presents from the tumor metabolome and what makes it unique compared with other approaches. Second, only a few MR1-restricted TCRs have been associated with tumors to date. Different TCRs have different transcriptomes and functions [7], which also could recognize various metabolic products that MR1 presents. This in turn, complicates the selection of a useful TCR to be used therapeutically. Third, *in vitro* functional assay and *in vivo* experiments verifying the effectivity and safety of MR1 function are limited. There is no clinical trial of the MR1-T cell for now. More fundamental studies on its effectiveness and safety are warranted before this approach can be used in clinical trials.

We recently identified a novel TCR clone of MR1-T cells that can kill AML cell lines (K562 and THP1) *in vitro* and kill AML cells from patients’ bone marrow *ex vivo*. However, the effectiveness and safety of these TCRs have yet to be assessed. MR1-T cells have the therapeutic potential but require further investigations.

## Abbreviations

2,4-DA-6-FP: 2,4-diamino-6-formylpteridine
3-FSA: 3-formylsalicylic acid
5-A-RU: 5-amino-6-d-ribitylaminouracil
5-OP-RU: 5-(2-oxopropylideneamino)-6-d-ribitylaminouracil
APC: antigen-presenting cell
CAR: chimeric antigen receptor
CD: cluster of differentiation
CDR: complementarity-determining region
CRS: cytokine release syndrome
DC: dendritic cell
GVHD: graft-versus-host disease
HLA: human leukocyte antigen
MAIT: mucosal-associated invariant T
MHC: major histocompatibility complex
MM: multiple myeloma
MR1: MHC class I-related
NK: natural killer
PAMP: pathogen-associated molecular pattern/pattern recognition receptor
TCR: T-cell receptor
TRA: T-cell receptor α locus
TRB: T-cell receptor β locus
